# 2,3,4,6-Tetra-*O*-acetyl-β-d-galacto­pyranosyl 2-(2,4-dichloro­anilino)-4,4-dimethyl-6-oxocyclo­hex-1-enecarbo­dithio­ate

**DOI:** 10.1107/S1600536809014743

**Published:** 2009-04-25

**Authors:** El Sayed H. El Ashry, Mohammed R. Amer, M. Raza Shah, Seik Weng Ng

**Affiliations:** a HEJ Research Institute of Chemistry, International Center for Chemical and Biological Sciences, University of Karachi, Karachi 75270, Pakistan; b Department of Chemistry, University of Malaya, 50603 Kuala Lumpur, Malaysia

## Abstract

The cyclo­hexene ring in the title compound, C_29_H_33_Cl_2_NO_10_S_2_, adopts an envelope conformation, with the C atom bearing the two methyl groups representing the flap. This atom deviates by 0.63 (1) Å from the plane through the other five ring atoms (r.m.s. deviation = 0.11 Å). The mol­ecular conformation is stabilized by an intra­molecular N—H⋯S hydrogen bond. The crystal studied was a non-merohedral twin, with a minor twin component of 29%.

## Related literature

For background to thio­glycosides, see: El Ashry *et al.* (2006 ▶, 2008 ▶), Haikel *et al.* (2003 ▶). For the deconvolution of non-merohedrally twinned diffraction data, see: Spek (2009 ▶).
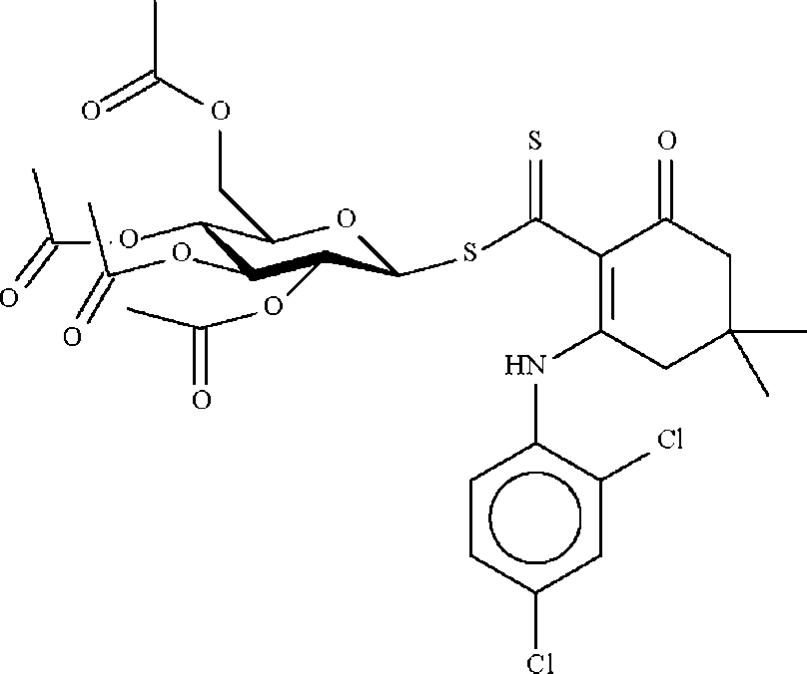


## Experimental

### 

#### Crystal data

C_29_H_33_Cl_2_NO_10_S_2_
*M* *_r_* = 690.58
Monoclinic, 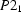

*a* = 13.8257 (4) Å
*b* = 8.7697 (3) Å
*c* = 14.0690 (4) Å
β = 106.486 (2)°
*V* = 1635.70 (9) Å^3^
*Z* = 2
Mo *K*α radiation
μ = 0.38 mm^−1^
*T* = 100 K
0.35 × 0.15 × 0.02 mm

#### Data collection

Bruker SMART APEX diffractometer
Absorption correction: multi-scan (*SADABS*; Sheldrick, 1996 ▶) *T*_min_ = 0.852, *T*_max_ = 0.992
15059 measured reflections
7402 independent reflections
5732 reflections with *I* > 2σ(*I*)
*R*_int_ = 0.065

#### Refinement

*R*[*F* ^2^ > 2σ(*F* ^2^)] = 0.092
*wR*(*F* ^2^) = 0.263
*S* = 1.09
7402 reflections
408 parameters
2 restraints
H atoms treated by a mixture of independent and constrained refinement
Δρ_max_ = 1.06 e Å^−3^
Δρ_min_ = −0.99 e Å^−3^
Absolute structure: Flack (1983 ▶), 3420 Friedel pairs
Flack parameter: 0.1 (2)

### 

Data collection: *APEX2* (Bruker, 2008 ▶); cell refinement: *SAINT* (Bruker, 2008 ▶); data reduction: *SAINT*; program(s) used to solve structure: *SHELXS97* (Sheldrick, 2008 ▶); program(s) used to refine structure: *SHELXL97* (Sheldrick, 2008 ▶); molecular graphics: *X-SEED* (Barbour, 2001 ▶); software used to prepare material for publication: *publCIF* (Westrip, 2009 ▶).

## Supplementary Material

Crystal structure: contains datablocks global, I. DOI: 10.1107/S1600536809014743/tk2430sup1.cif

Structure factors: contains datablocks I. DOI: 10.1107/S1600536809014743/tk2430Isup2.hkl

Additional supplementary materials:  crystallographic information; 3D view; checkCIF report

## Figures and Tables

**Table 1:** Hydrogen-bond geometry (Å, °)

*D*—H⋯*A*	*D*—H	H⋯*A*	*D*⋯*A*	*D*—H⋯*A*
N1—H1⋯S2	0.88 (1)	2.07 (5)	2.882 (6)	152 (9)

**Fig. 1.**
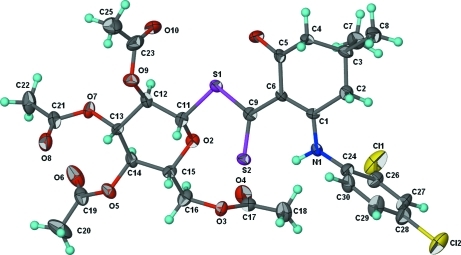
Thermal ellipsoid plot (Barbour, 2001) plot of C29H33Cl2NOS2 at the 70% probability level; hydrogen atoms are drawn as spheres of arbitrary radius.

## supplementary crystallographic information

### Experimental

A cooled (283 K) solution of (2,4-dichloroanilino)-5,5-dimethyl-cyclohex-2-en-1-one (0.1 mol) and sodium hydroxide (0.4 g) in DMSO (20 ml) and water (1 ml) was treated with carbon disulfide (0.3 mol). After 20 min, 2,3,4,6-tetra-*O*-acetyl-α-*D*-galactopyranosyl bromide (0.12 mol) was added and the reaction mixture was left for 24 h. Water (200 ml) was added and the mixture acidified with 10% hydrochloric acid. The product was purified on by silica-gel column-chromatography to give yellow crystals that were further crystallized from methanol (m.p. 480 K).

### Refinement

Carbon-bound H-atoms were placed in calculated positions (C—H 0.95 to 1.00 Å) and were included in the refinement in the riding model approximation, with *U*(H) set to 1.2 to 1.5*U*(C). The amino H-atom was located in a difference Fourier map and was refined with a distance restraint of N–H 0.88±0.01 Å; its displacement factor was freely refined.

The structure initally refined to *R* = 0.118 but the displacement factors and bond dimensions for all atoms were normal. Subsequent analysis showed the structure to be a non-merohedral twin. *PLATON* (Spek, 2009) split the reflection data by the matrix (-1 0 0, 0 - 1 0, 0.578 0 1). The minor twin component refined to 0.287.

The final difference Fourier map had a large peak at 1.5 Å from H4a.

### Crystal data

**Table:**

C_29_H_33_Cl_2_NO_10_S_2_	*F*(000) = 720
*M**_r_* = 690.58	*D*_x_ = 1.402 Mg m^−^^3^
Monoclinic, *P*2_1_	Mo *K*α radiation, λ = 0.71073 Å
Hall symbol: P 2yb	Cell parameters from 2620 reflections
*a* = 13.8257 (4) Å	θ = 2.8–21.7°
*b* = 8.7697 (3) Å	µ = 0.38 mm^−^^1^
*c* = 14.0690 (4) Å	*T* = 100 K
β = 106.486 (2)°	Plate, orange
*V* = 1635.70 (9) Å^3^	0.35 × 0.15 × 0.02 mm
*Z* = 2	

### Data collection

**Table:**

Bruker SMART APEX diffractometer	7402 independent reflections
Radiation source: fine-focus sealed tube	5732 reflections with *I* > 2σ(*I*)
graphite	*R*_int_ = 0.065
ω scans	θ_max_ = 27.5°, θ_min_ = 1.8°
Absorption correction: multi-scan (*SADABS*; Sheldrick, 1996)	*h* = −17→17
*T*_min_ = 0.852, *T*_max_ = 0.992	*k* = −11→11
15059 measured reflections	*l* = −18→18

### Refinement

**Table:**

Refinement on *F*^2^	Secondary atom site location: difference Fourier map
Least-squares matrix: full	Hydrogen site location: inferred from neighbouring sites
*R*[*F*^2^ > 2σ(*F*^2^)] = 0.092	H atoms treated by a mixture of independent and constrained refinement
*wR*(*F*^2^) = 0.263	*w* = 1/[σ^2^(*F*_o_^2^) + (0.1355*P*)^2^ + 3.0358*P*] where *P* = (*F*_o_^2^ + 2*F*_c_^2^)/3
*S* = 1.09	(Δ/σ)_max_ = 0.001
7402 reflections	Δρ_max_ = 1.06 e Å^−^^3^
408 parameters	Δρ_min_ = −0.99 e Å^−^^3^
2 restraints	Absolute structure: Flack (1983), 3420 Friedel pairs
Primary atom site location: structure-invariant direct methods	Flack parameter: 0.1 (2)

### Fractional atomic coordinates and isotropic or equivalent isotropic displacement parameters (Å^2^)

**Table:**

	*x*	*y*	*z*	*U*_iso_*/*U*_eq_	
S1	0.78612 (13)	0.4998 (2)	0.07646 (13)	0.0206 (4)	
S2	0.70294 (13)	0.2505 (2)	−0.06603 (14)	0.0213 (4)	
Cl1	0.7299 (2)	0.4115 (3)	−0.39454 (16)	0.0473 (6)	
Cl2	0.8139 (2)	−0.0943 (3)	−0.57216 (16)	0.0449 (6)	
O1	0.9601 (5)	0.5765 (7)	0.0630 (4)	0.0350 (14)	
O2	0.5862 (4)	0.4620 (5)	0.0353 (4)	0.0192 (10)	
O3	0.4399 (4)	0.3463 (6)	−0.1291 (4)	0.0214 (11)	
O4	0.3754 (5)	0.5700 (6)	−0.1994 (4)	0.0327 (13)	
O5	0.4186 (4)	0.2941 (6)	0.1614 (4)	0.0238 (11)	
O6	0.3331 (5)	0.4616 (7)	0.2260 (5)	0.0368 (15)	
O7	0.5631 (4)	0.5043 (6)	0.3097 (3)	0.0216 (10)	
O8	0.5287 (5)	0.2970 (7)	0.3894 (4)	0.0328 (14)	
O9	0.7568 (4)	0.4371 (6)	0.2862 (4)	0.0245 (11)	
O10	0.8313 (5)	0.6649 (7)	0.2879 (4)	0.0380 (15)	
N1	0.8206 (4)	0.2397 (8)	−0.2053 (4)	0.0204 (12)	
H1	0.774 (5)	0.218 (11)	−0.176 (6)	0.03 (3)*	
C1	0.8882 (5)	0.3347 (8)	−0.1512 (5)	0.0167 (13)	
C2	0.9760 (5)	0.3714 (8)	−0.1929 (5)	0.0211 (15)	
H2A	1.0162	0.2773	−0.1904	0.025*	
H2B	0.9479	0.3992	−0.2636	0.025*	
C3	1.0468 (5)	0.4979 (9)	−0.1419 (6)	0.0231 (14)	
C4	1.0629 (5)	0.4783 (9)	−0.0316 (5)	0.0209 (15)	
H4A	1.0920	0.3764	−0.0108	0.025*	
H4B	1.1113	0.5560	0.0048	0.025*	
C5	0.9649 (5)	0.4947 (9)	−0.0061 (5)	0.0224 (15)	
C6	0.8785 (5)	0.4039 (9)	−0.0634 (5)	0.0199 (14)	
C7	1.0011 (7)	0.6559 (9)	−0.1783 (7)	0.0316 (18)	
H7A	0.9774	0.6565	−0.2509	0.047*	
H7B	1.0527	0.7348	−0.1556	0.047*	
H7C	0.9443	0.6764	−0.1514	0.047*	
C8	1.1460 (6)	0.4828 (11)	−0.1689 (7)	0.0336 (19)	
H8A	1.1726	0.3792	−0.1537	0.050*	
H8B	1.1949	0.5565	−0.1304	0.050*	
H8C	1.1341	0.5029	−0.2398	0.050*	
C9	0.7936 (5)	0.3796 (8)	−0.0239 (5)	0.0189 (14)	
C11	0.6779 (5)	0.4207 (8)	0.1073 (5)	0.0181 (13)	
H11	0.6838	0.3071	0.1115	0.022*	
C12	0.6713 (5)	0.4842 (9)	0.2059 (5)	0.0208 (14)	
H12	0.6652	0.5979	0.2031	0.025*	
C13	0.5805 (5)	0.4115 (8)	0.2314 (5)	0.0171 (13)	
H13	0.5976	0.3051	0.2559	0.021*	
C14	0.4841 (5)	0.4106 (9)	0.1444 (5)	0.0207 (14)	
H14	0.4501	0.5123	0.1375	0.025*	
C15	0.5079 (5)	0.3665 (8)	0.0481 (5)	0.0185 (14)	
H15	0.5297	0.2574	0.0513	0.022*	
C16	0.4140 (6)	0.3897 (9)	−0.0387 (5)	0.0252 (16)	
H16A	0.3581	0.3254	−0.0304	0.030*	
H16B	0.3924	0.4977	−0.0425	0.030*	
C17	0.4192 (6)	0.4505 (9)	−0.2029 (6)	0.0245 (16)	
C18	0.4605 (7)	0.3996 (10)	−0.2836 (6)	0.0339 (18)	
H18A	0.5175	0.4648	−0.2855	0.051*	
H18B	0.4079	0.4066	−0.3471	0.051*	
H18C	0.4834	0.2937	−0.2719	0.051*	
C19	0.3470 (6)	0.3317 (10)	0.2036 (6)	0.0281 (17)	
C20	0.2888 (8)	0.1967 (12)	0.2189 (9)	0.052 (3)	
H20A	0.2653	0.2126	0.2777	0.077*	
H20B	0.3319	0.1061	0.2285	0.077*	
H20C	0.2306	0.1823	0.1607	0.077*	
C21	0.5345 (6)	0.4330 (9)	0.3822 (6)	0.0252 (16)	
C22	0.5020 (7)	0.5445 (11)	0.4462 (7)	0.037 (2)	
H22A	0.5078	0.4982	0.5110	0.056*	
H22B	0.4318	0.5738	0.4150	0.056*	
H22C	0.5451	0.6352	0.4550	0.056*	
C23	0.8296 (6)	0.5446 (11)	0.3232 (7)	0.035 (2)	
C24	0.8238 (5)	0.1667 (9)	−0.2938 (5)	0.0215 (15)	
C25	0.9019 (7)	0.4892 (15)	0.4157 (7)	0.050 (3)	
H25A	0.9698	0.5260	0.4192	0.075*	
H25B	0.9018	0.3774	0.4164	0.075*	
H25C	0.8821	0.5275	0.4729	0.075*	
C26	0.7794 (6)	0.2291 (9)	−0.3864 (6)	0.0257 (16)	
C27	0.7763 (7)	0.1509 (11)	−0.4737 (6)	0.0301 (17)	
H27	0.7479	0.1967	−0.5367	0.036*	
C28	0.8154 (7)	0.0059 (11)	−0.4659 (6)	0.0321 (18)	
C29	0.8613 (7)	−0.0591 (9)	−0.3748 (6)	0.0320 (19)	
H29	0.8900	−0.1581	−0.3712	0.038*	
C30	0.8655 (6)	0.0206 (9)	−0.2893 (6)	0.0253 (16)	
H30	0.8970	−0.0242	−0.2266	0.030*	

### Atomic displacement parameters (Å^2^)

**Table:**

	*U*^11^	*U*^22^	*U*^33^	*U*^12^	*U*^13^	*U*^23^
S1	0.0217 (8)	0.0199 (8)	0.0235 (8)	−0.0054 (7)	0.0115 (7)	−0.0049 (7)
S2	0.0182 (8)	0.0227 (8)	0.0256 (9)	−0.0056 (7)	0.0107 (7)	−0.0072 (7)
Cl1	0.0630 (15)	0.0380 (12)	0.0311 (11)	0.0245 (12)	−0.0025 (10)	−0.0023 (9)
Cl2	0.0575 (14)	0.0491 (14)	0.0272 (10)	0.0067 (12)	0.0103 (10)	−0.0145 (10)
O1	0.037 (3)	0.045 (3)	0.028 (3)	−0.023 (3)	0.017 (3)	−0.019 (3)
O2	0.018 (2)	0.019 (2)	0.019 (2)	0.0023 (19)	0.004 (2)	0.0006 (18)
O3	0.027 (3)	0.022 (3)	0.016 (2)	0.001 (2)	0.007 (2)	−0.0001 (19)
O4	0.044 (4)	0.021 (3)	0.038 (3)	0.003 (3)	0.019 (3)	0.002 (2)
O5	0.017 (2)	0.026 (3)	0.032 (3)	−0.001 (2)	0.014 (2)	0.000 (2)
O6	0.034 (3)	0.029 (3)	0.056 (4)	0.009 (2)	0.028 (3)	0.011 (3)
O7	0.027 (3)	0.023 (2)	0.017 (2)	0.005 (2)	0.011 (2)	−0.001 (2)
O8	0.048 (4)	0.031 (3)	0.024 (3)	−0.005 (3)	0.016 (3)	0.002 (2)
O9	0.019 (3)	0.027 (3)	0.026 (3)	−0.004 (2)	0.004 (2)	−0.002 (2)
O10	0.051 (4)	0.035 (3)	0.030 (3)	−0.022 (3)	0.014 (3)	−0.014 (3)
N1	0.016 (3)	0.028 (3)	0.020 (3)	−0.005 (3)	0.010 (2)	−0.003 (2)
C1	0.014 (3)	0.016 (3)	0.019 (3)	−0.002 (3)	0.003 (3)	−0.001 (3)
C2	0.020 (3)	0.023 (4)	0.022 (3)	0.003 (3)	0.008 (3)	−0.001 (3)
C3	0.016 (3)	0.026 (4)	0.027 (4)	−0.006 (3)	0.005 (3)	0.000 (3)
C4	0.013 (3)	0.028 (4)	0.021 (3)	−0.007 (3)	0.005 (3)	−0.003 (3)
C5	0.025 (4)	0.025 (3)	0.023 (3)	−0.006 (3)	0.016 (3)	−0.007 (3)
C6	0.018 (3)	0.024 (4)	0.019 (3)	−0.008 (3)	0.008 (3)	−0.002 (3)
C7	0.038 (5)	0.022 (4)	0.038 (5)	0.000 (4)	0.016 (4)	−0.001 (3)
C8	0.029 (4)	0.040 (5)	0.039 (5)	−0.010 (4)	0.022 (4)	−0.013 (4)
C9	0.018 (3)	0.017 (3)	0.020 (3)	0.003 (3)	0.004 (3)	0.004 (3)
C11	0.016 (3)	0.021 (3)	0.016 (3)	0.005 (3)	0.002 (3)	0.002 (3)
C12	0.022 (3)	0.022 (4)	0.021 (3)	0.000 (3)	0.011 (3)	0.003 (3)
C13	0.018 (3)	0.019 (3)	0.015 (3)	−0.002 (3)	0.005 (3)	−0.004 (3)
C14	0.023 (4)	0.019 (3)	0.025 (4)	0.002 (3)	0.015 (3)	0.001 (3)
C15	0.010 (3)	0.025 (4)	0.020 (3)	0.002 (3)	0.003 (3)	−0.005 (3)
C16	0.025 (4)	0.029 (4)	0.019 (3)	0.006 (3)	0.003 (3)	0.000 (3)
C17	0.025 (4)	0.024 (4)	0.024 (4)	−0.004 (3)	0.007 (3)	−0.003 (3)
C18	0.040 (5)	0.032 (4)	0.033 (4)	0.003 (4)	0.015 (4)	0.008 (4)
C19	0.020 (4)	0.038 (5)	0.028 (4)	0.004 (3)	0.011 (3)	0.007 (3)
C20	0.030 (5)	0.048 (6)	0.085 (8)	−0.013 (4)	0.030 (5)	−0.003 (6)
C21	0.024 (4)	0.031 (4)	0.023 (4)	0.002 (3)	0.011 (3)	0.005 (3)
C22	0.045 (5)	0.041 (5)	0.037 (5)	0.010 (4)	0.030 (4)	0.000 (4)
C23	0.029 (4)	0.046 (6)	0.033 (5)	−0.006 (4)	0.014 (4)	−0.022 (4)
C24	0.018 (3)	0.028 (4)	0.018 (3)	−0.003 (3)	0.006 (3)	−0.005 (3)
C25	0.037 (5)	0.074 (7)	0.034 (5)	−0.025 (5)	0.002 (4)	0.000 (5)
C26	0.022 (4)	0.030 (4)	0.025 (4)	0.005 (3)	0.006 (3)	−0.004 (3)
C27	0.032 (4)	0.039 (4)	0.017 (4)	0.002 (4)	0.004 (3)	−0.008 (3)
C28	0.036 (4)	0.038 (5)	0.024 (4)	0.001 (4)	0.011 (3)	−0.006 (4)
C29	0.046 (5)	0.023 (4)	0.029 (4)	0.017 (4)	0.015 (4)	0.003 (3)
C30	0.035 (4)	0.022 (4)	0.023 (4)	0.005 (3)	0.013 (3)	0.004 (3)

### Geometric parameters (Å, °)

**Table:**

S1—C9	1.788 (7)	C8—H8B	0.9800
S1—C11	1.811 (7)	C8—H8C	0.9800
S2—C9	1.668 (7)	C11—C12	1.521 (9)
Cl1—C26	1.730 (8)	C11—H11	1.0000
Cl2—C28	1.729 (8)	C12—C13	1.539 (9)
O1—C5	1.226 (9)	C12—H12	1.0000
O2—C15	1.420 (8)	C13—C14	1.532 (10)
O2—C11	1.426 (8)	C13—H13	1.0000
O3—C17	1.351 (9)	C14—C15	1.532 (9)
O3—C16	1.466 (9)	C14—H14	1.0000
O4—C17	1.219 (10)	C15—C16	1.523 (9)
O5—C19	1.332 (9)	C15—H15	1.0000
O5—C14	1.429 (9)	C16—H16A	0.9900
O6—C19	1.212 (10)	C16—H16B	0.9900
O7—C21	1.348 (9)	C17—C18	1.477 (11)
O7—C13	1.444 (8)	C18—H18A	0.9800
O8—C21	1.201 (10)	C18—H18B	0.9800
O9—C23	1.369 (10)	C18—H18C	0.9800
O9—C12	1.445 (9)	C19—C20	1.481 (12)
O10—C23	1.169 (12)	C20—H20A	0.9800
N1—C1	1.321 (9)	C20—H20B	0.9800
N1—C24	1.411 (9)	C20—H20C	0.9800
N1—H1	0.881 (10)	C21—C22	1.483 (11)
C1—C6	1.415 (10)	C22—H22A	0.9800
C1—C2	1.525 (10)	C22—H22B	0.9800
C2—C3	1.519 (10)	C22—H22C	0.9800
C2—H2A	0.9900	C23—C25	1.481 (14)
C2—H2B	0.9900	C24—C26	1.385 (11)
C3—C4	1.514 (10)	C24—C30	1.400 (11)
C3—C8	1.529 (10)	C25—H25A	0.9800
C3—C7	1.548 (11)	C25—H25B	0.9800
C4—C5	1.504 (9)	C25—H25C	0.9800
C4—H4A	0.9900	C26—C27	1.398 (11)
C4—H4B	0.9900	C27—C28	1.374 (13)
C5—C6	1.471 (10)	C27—H27	0.9500
C6—C9	1.451 (9)	C28—C29	1.381 (12)
C7—H7A	0.9800	C29—C30	1.379 (11)
C7—H7B	0.9800	C29—H29	0.9500
C7—H7C	0.9800	C30—H30	0.9500
C8—H8A	0.9800		
			
C9—S1—C11	101.5 (3)	C15—C14—C13	110.5 (5)
C15—O2—C11	108.4 (5)	O5—C14—H14	110.6
C17—O3—C16	116.2 (6)	C15—C14—H14	110.6
C19—O5—C14	118.9 (6)	C13—C14—H14	110.6
C21—O7—C13	117.6 (6)	O2—C15—C16	109.1 (6)
C23—O9—C12	116.7 (7)	O2—C15—C14	109.0 (5)
C1—N1—C24	127.0 (6)	C16—C15—C14	109.1 (5)
C1—N1—H1	111 (6)	O2—C15—H15	109.9
C24—N1—H1	122 (6)	C16—C15—H15	109.9
N1—C1—C6	123.1 (6)	C14—C15—H15	109.9
N1—C1—C2	114.9 (6)	O3—C16—C15	107.4 (6)
C6—C1—C2	121.9 (6)	O3—C16—H16A	110.2
C3—C2—C1	116.7 (6)	C15—C16—H16A	110.2
C3—C2—H2A	108.1	O3—C16—H16B	110.2
C1—C2—H2A	108.1	C15—C16—H16B	110.2
C3—C2—H2B	108.1	H16A—C16—H16B	108.5
C1—C2—H2B	108.1	O4—C17—O3	123.4 (7)
H2A—C2—H2B	107.3	O4—C17—C18	126.5 (8)
C4—C3—C2	106.8 (6)	O3—C17—C18	110.1 (7)
C4—C3—C8	111.3 (6)	C17—C18—H18A	109.5
C2—C3—C8	108.8 (6)	C17—C18—H18B	109.5
C4—C3—C7	111.6 (7)	H18A—C18—H18B	109.5
C2—C3—C7	110.5 (6)	C17—C18—H18C	109.5
C8—C3—C7	107.8 (7)	H18A—C18—H18C	109.5
C5—C4—C3	110.7 (6)	H18B—C18—H18C	109.5
C5—C4—H4A	109.5	O6—C19—O5	122.8 (7)
C3—C4—H4A	109.5	O6—C19—C20	125.5 (8)
C5—C4—H4B	109.5	O5—C19—C20	111.8 (8)
C3—C4—H4B	109.5	C19—C20—H20A	109.5
H4A—C4—H4B	108.1	C19—C20—H20B	109.5
O1—C5—C6	122.3 (6)	H20A—C20—H20B	109.5
O1—C5—C4	120.0 (7)	C19—C20—H20C	109.5
C6—C5—C4	117.6 (6)	H20A—C20—H20C	109.5
C1—C6—C9	124.0 (6)	H20B—C20—H20C	109.5
C1—C6—C5	116.6 (6)	O8—C21—O7	124.4 (7)
C9—C6—C5	119.3 (6)	O8—C21—C22	124.4 (7)
C3—C7—H7A	109.5	O7—C21—C22	111.0 (7)
C3—C7—H7B	109.5	C21—C22—H22A	109.5
H7A—C7—H7B	109.5	C21—C22—H22B	109.5
C3—C7—H7C	109.5	H22A—C22—H22B	109.5
H7A—C7—H7C	109.5	C21—C22—H22C	109.5
H7B—C7—H7C	109.5	H22A—C22—H22C	109.5
C3—C8—H8A	109.5	H22B—C22—H22C	109.5
C3—C8—H8B	109.5	O10—C23—O9	124.2 (9)
H8A—C8—H8B	109.5	O10—C23—C25	125.4 (9)
C3—C8—H8C	109.5	O9—C23—C25	110.3 (8)
H8A—C8—H8C	109.5	C26—C24—C30	118.1 (7)
H8B—C8—H8C	109.5	C26—C24—N1	122.3 (7)
C6—C9—S2	124.9 (5)	C30—C24—N1	119.4 (7)
C6—C9—S1	116.8 (5)	C23—C25—H25A	109.5
S2—C9—S1	118.4 (4)	C23—C25—H25B	109.5
O2—C11—C12	106.4 (5)	H25A—C25—H25B	109.5
O2—C11—S1	111.1 (5)	C23—C25—H25C	109.5
C12—C11—S1	110.2 (5)	H25A—C25—H25C	109.5
O2—C11—H11	109.7	H25B—C25—H25C	109.5
C12—C11—H11	109.7	C24—C26—C27	121.9 (7)
S1—C11—H11	109.7	C24—C26—Cl1	119.3 (6)
O9—C12—C11	111.2 (6)	C27—C26—Cl1	118.8 (6)
O9—C12—C13	103.8 (5)	C28—C27—C26	118.1 (8)
C11—C12—C13	109.0 (6)	C28—C27—H27	121.0
O9—C12—H12	110.9	C26—C27—H27	121.0
C11—C12—H12	110.9	C27—C28—C29	121.5 (7)
C13—C12—H12	110.9	C27—C28—Cl2	119.5 (7)
O7—C13—C14	108.5 (5)	C29—C28—Cl2	118.8 (7)
O7—C13—C12	105.6 (5)	C30—C29—C28	119.7 (7)
C14—C13—C12	113.5 (5)	C30—C29—H29	120.2
O7—C13—H13	109.7	C28—C29—H29	120.2
C14—C13—H13	109.7	C29—C30—C24	120.6 (7)
C12—C13—H13	109.7	C29—C30—H30	119.7
O5—C14—C15	105.8 (6)	C24—C30—H30	119.7
O5—C14—C13	108.6 (6)		
			
C24—N1—C1—C6	179.5 (7)	O9—C12—C13—C14	−165.2 (6)
C24—N1—C1—C2	2.5 (11)	C11—C12—C13—C14	−46.6 (8)
N1—C1—C2—C3	170.9 (6)	C19—O5—C14—C15	−146.1 (6)
C6—C1—C2—C3	−6.1 (10)	C19—O5—C14—C13	95.3 (7)
C1—C2—C3—C4	40.8 (8)	O7—C13—C14—O5	−85.2 (6)
C1—C2—C3—C8	161.1 (7)	C12—C13—C14—O5	157.9 (6)
C1—C2—C3—C7	−80.7 (8)	O7—C13—C14—C15	159.2 (6)
C2—C3—C4—C5	−61.7 (8)	C12—C13—C14—C15	42.2 (8)
C8—C3—C4—C5	179.6 (7)	C11—O2—C15—C16	−170.2 (5)
C7—C3—C4—C5	59.1 (8)	C11—O2—C15—C14	70.8 (7)
C3—C4—C5—O1	−132.3 (8)	O5—C14—C15—O2	−170.0 (5)
C3—C4—C5—C6	50.7 (9)	C13—C14—C15—O2	−52.6 (8)
N1—C1—C6—C9	−0.5 (12)	O5—C14—C15—C16	71.0 (7)
C2—C1—C6—C9	176.3 (7)	C13—C14—C15—C16	−171.6 (6)
N1—C1—C6—C5	174.0 (7)	C17—O3—C16—C15	−127.5 (7)
C2—C1—C6—C5	−9.1 (10)	O2—C15—C16—O3	61.0 (7)
O1—C5—C6—C1	169.7 (8)	C14—C15—C16—O3	−180.0 (6)
C4—C5—C6—C1	−13.3 (10)	C16—O3—C17—O4	−4.9 (11)
O1—C5—C6—C9	−15.4 (12)	C16—O3—C17—C18	173.0 (6)
C4—C5—C6—C9	161.5 (7)	C14—O5—C19—O6	2.1 (12)
C1—C6—C9—S2	8.6 (11)	C14—O5—C19—C20	−177.9 (7)
C5—C6—C9—S2	−165.8 (6)	C13—O7—C21—O8	4.9 (11)
C1—C6—C9—S1	−170.6 (6)	C13—O7—C21—C22	−169.7 (7)
C5—C6—C9—S1	15.0 (9)	C12—O9—C23—O10	−7.7 (11)
C11—S1—C9—C6	−175.6 (5)	C12—O9—C23—C25	169.7 (7)
C11—S1—C9—S2	5.2 (5)	C1—N1—C24—C26	−92.1 (10)
C15—O2—C11—C12	−75.1 (6)	C1—N1—C24—C30	93.4 (10)
C15—O2—C11—S1	164.9 (4)	C30—C24—C26—C27	−0.5 (12)
C9—S1—C11—O2	−74.5 (5)	N1—C24—C26—C27	−175.1 (7)
C9—S1—C11—C12	167.7 (5)	C30—C24—C26—Cl1	−178.4 (6)
C23—O9—C12—C11	104.5 (7)	N1—C24—C26—Cl1	7.0 (10)
C23—O9—C12—C13	−138.5 (6)	C24—C26—C27—C28	2.4 (13)
O2—C11—C12—O9	174.8 (5)	Cl1—C26—C27—C28	−179.6 (7)
S1—C11—C12—O9	−64.6 (6)	C26—C27—C28—C29	−3.3 (13)
O2—C11—C12—C13	61.0 (7)	C26—C27—C28—Cl2	−179.5 (7)
S1—C11—C12—C13	−178.4 (5)	C27—C28—C29—C30	2.2 (14)
C21—O7—C13—C14	95.4 (7)	Cl2—C28—C29—C30	178.4 (7)
C21—O7—C13—C12	−142.7 (6)	C28—C29—C30—C24	−0.1 (13)
O9—C12—C13—O7	76.2 (6)	C26—C24—C30—C29	−0.7 (11)
C11—C12—C13—O7	−165.3 (5)	N1—C24—C30—C29	174.1 (7)

### Hydrogen-bond geometry (Å, °)

**Table:**

*D*—H···*A*	*D*—H	H···*A*	*D*···*A*	*D*—H···*A*
N1—H1···S2	0.88 (1)	2.07 (5)	2.882 (6)	152 (9)
